# On Line Disaster Response Community: People as Sensors of High Magnitude Disasters Using Internet GIS

**DOI:** 10.3390/s8053037

**Published:** 2008-05-06

**Authors:** Melinda Laituri, Kris Kodrich

**Affiliations:** Colorado State University, Warner College of Natural Resources, 970-491-0292, FRWS 1472, Fort Collins, Colorado 80423, USA

**Keywords:** disaster, internet GIS, Hurricane Katrina, tsunami, media, on-line

## Abstract

The Indian Ocean tsunami (2004) and Hurricane Katrina (2005) reveal the coming of age of the on-line disaster response community. Due to the integration of key geospatial technologies (remote sensing - RS, geographic information systems - GIS, global positioning systems – GPS) and the Internet, on-line disaster response communities have grown. They include the traditional aspects of disaster preparedness, response, recovery, mitigation, and policy as facilitated by governmental agencies and relief response organizations. However, the contribution from the public via the Internet has changed significantly. The on-line disaster response community includes several key characteristics: the ability to donate money quickly and efficiently due to improved Internet security and reliable donation sites; a computer-savvy segment of the public that creates blogs, uploads pictures, and disseminates information – oftentimes faster than government agencies, and message boards to create interactive information exchange in seeking family members and identifying shelters. A critical and novel occurrence is the development of “people as sensors” - networks of government, NGOs, private companies, and the public - to build rapid response databases of the disaster area for various aspects of disaster relief and response using geospatial technologies. This paper examines these networks, their products, and their future potential.

## Introduction

Multiple, large scale disastrous events continually occur. The 2004 Indian Ocean tsunami devastated a dozen countries, killing more than 275,000, with thousands missing, and leaving millions homeless. In September 2005, Hurricane Katrina, a category three storm, caused significant damage in several US cities, including New Orleans, LA, Mobile, AL, and Gulfport, MS. Several hundred people were killed and thousands left homeless (http://www.whitehouse.gov/reports/katrina-lessons-learned/chapter1.html). In October 2005 an earthquake of magnitude 7.6 occurred in Kashmir, Pakistan. Fatalities exceed 40,000 making it the most fatal earthquake ever to occur in the Indian subcontinent. Thousands were vulnerable to exposure due to the onset of winter and loss of homes [52]. In 2006, Europe experienced record floods along the Danube causing severe damage and evacuations in Hungary, Romania, Bulgaria and Serbia [47]. The 2006/2007 typhoon season in Malaysia, Singapore and Indonesia resulted in 400,000 people being displaced [40]. The 2007 California wildfires resulted in the loss of nearly 2200 homes and over $1 billion dollars in damages (http://www.cbsnews.com/stories/2007/10/24/national/main3401265.shtml).

Along with increasing global population, the magnitude and frequency of disasters appear to be increasing [19]. Governments, NGOs and disaster response agencies must manage disaster processes and consequences in order to mitigate against, prepare for, respond to and recover from disasters. Effective disaster management and response demands rapid utilization of information and data from many sources. The ability to seamlessly integrate and distribute digital data into spatially explicit formats for rapid assessment and analysis during and post disaster is improving. The suite of geospatial technologies (GIS, RS, GPS and the Internet) are playing an increasingly important role in disaster management, response and preparation. Remotely sensed data provides earth observation information that can be displayed in conjunction with other digital, spatial data in a geographic information system for analysis. Use of the Internet enhances the delivery of geospatial technologies to a larger audience, facilitating the exchange of information, and increasing the speed of communication. In 2007, The National Academy of Sciences released a report describing the state-of-the-art with regard to geospatial data and tools for emergency management focusing on the emergency management community made up of governmental agencies, first responders in the field, and other coordinating centers and the challenges they face in using geospatial information [33]. An important addition to this group of responders are the ad hoc citizen groups, local victims of the event and their families, and computer-based disaster response volunteers that collect and disseminate data in the form of text, blogs, video, pictures, and maps.

Disasters are complex events “concentrated in time and space, in which a community experiences severe damage and disruption of its essential functions, accompanied by widespread human, material or environmental losses, which often exceed the ability of the community to cope without external assistance” [48]. One form of external assistance is the online disaster-response community (ODRC) that is comprised of formal and informal networks of people acting as sensors collecting, processing, and delivering information where it is needed. In research examining the new media technologies on charitable giving after the Indian Ocean tsunami and Hurricanes Katrina and Rita, Kodrich and Laituri defined two tiers of the ODRC:

- The first tier is the formal network of national and international disaster awareness and humanitarian aid programs of the disaster response organizations and governmental agencies. Their task is ongoing in terms of raising public awareness about disasters and raising monies to support their initiatives. They function on a continuous basis and facilitate communication, networking, and assistance in response to specific disasters.
- The second tier involves the immediate disaster response when a disaster occurs. It is multidimensional and facilitated by a wide array of participants: the media, the general public, concerned and interested parties (aid groups, family, government). This tier is largely transitory, locally or community based, built on a particular event that occurs in a specific geographic area and includes online “first responders of the wired world” [7]. This tier is composed of the formal networks such as online news reporting, emergency information services, and mechanisms for online donations. Increasingly, informal social networks of communication have developed that provide first hand accounts of the disaster, posting of pictures and videos, blogs, and chatrooms that assist in locating resources that often link to the formal emergency response network [24].

A third tier can be considered that includes the use of geospatial technologies to enhance and improve the transformation of data into information and the dissemination of geospatial information for disaster response.

- This tier is evolving as an ongoing presence that can provide information throughout and after the disaster event, monitor relief efforts, and model response scenarios. A key characteristic of this third tier is the enhanced use of geospatial technologies and the interaction between formal and informal socio-technological networks on the Internet. Participatory responses via the Internet during the disaster reveal the contributions from intensive contacts between organizationally distinct groups that create networks facilitating the use of Internet GIS for disaster management, monitoring, modeling, response, and relief.

This paper focuses on the development and use of Internet GIS by the ODRC with particular emphasis on participatory responses and activities from novel networks of users during and post-disaster. Internet GIS is the network-based geographic information services that can utilize wired or wireless internet to access geographic information, spatial analysis tools and GIS web services and allow for broad dissemination of data and analysis results [37]. The integration of these geospatial tools and datasets that allow for Internet access has become a key factor during and after a disaster event. The Indian Ocean tsunami and Hurricane Katrina reveal important advances that have occurred via the use of Internet GIS. For example, the disaster of the Indian Ocean tsunami revealed the ability to quickly provide remotely sensed images both before and after the event that showed the extent of damage. This occurred through partnerships between software vendors, Internet service providers, and remote sensing companies (http://www.dmsolutions.ca/solutions/tsunami.html). However, these disasters as well as the Kashmiri earthquake (2005) expose the inherent shortcomings of Internet GIS for disaster response. The failure of FEMA in the aftermath of Hurricane Katrina resulted in numerous individuals to respond via establishing donation sites, message boards, and maps of emergency aid sites. The notion of “people as sensors” - people collecting information to aid in the recovery process and posting this information for broad dissemination outside of the established traditional channels of emergency response is an aspect of disaster response that needs to be examined. Identifying and understanding the lessons from these key events may facilitate a better understanding of disasters and the role of geospatial technologies to improve our management of and response to them.

## Cyberspace communities, Disasters, and Participation

Since the World Wide Web gained popularity in the mid-1990's, it has brought people together creating virtual communities in cyberspace [2, 22, 43]. Virtual communities are where members are not necessarily physically close to each other, have common interests, and receive, create and exchange information that are not tightly linked to their geographic location [2, 43]. These communities occur when people interact for enough time and with enough feeling and sense of participation to form webs of personal interaction [41]. People form personal relationships and engage in social interactions in virtual space creating networks of communication [22, 41].

Electronic media are critical to linking the socio-physical world with the symbolic world [44]. The Internet and mobile technologies have been particularly effective in linking disasters to written accounts, photographs and web diaries, connecting to a larger audience that can vicariously participate in the disaster. Novel uses of satellite imagery, GIS, and internet technologies are important in translating disaster information to other audiences. In research on the Gujarat earthquake of 2001, Kodrich and Laituri identified the following characteristics with regard to online technology, communication, and disasters:

- Online media facilitate communication among families, friends and others, both inside and outside the country in which the disaster occurs, during a disaster.
- The messages and images carried about a disaster convey a sense of urgency to the world.
- The Internet enhances the ability for interactive communication of relevant information quickly and efficiently, provided people have the means to access the technology.
- Different forms of media interact to fuel news stories and information dissemination. The Internet, online media and blogs work in concert, remixing and reamplifying information.
- Web sites, wikis, and blogs can offer immediate assistance and assurance to a community, such as information on relief efforts, locations of impacted areas, potential dangers, shelter locations, donations, and ways to assist.
- The technological capabilities of the Internet make it an ideal tool for reporting accurate (and inaccurate) information, very quickly, and distributing it to the largest audience possible.
- Different types of information can be made rapidly available that depict the geographic extent of the event and satellite images provide a bird's eye view of the location.
- People living around the world have the opportunity to learn about the human tragedy that results from a disaster (provided they have Internet access) and this fosters a sense of global community [25].

Internet access and use has facilitated the development of citizen or participatory journalism. Citizen journalism integrates open source capabilities for blogging, wiki development, mobile phone use and Internet GIS to produce and disseminate information by amateurs, technologists, and activists [31]. Since 1999, the phenomenon of blogging has led to citizen participation in events where their views and skills are initially shared informally and serendipitously [10]. Where disasters are concerned, there has been a similar informal development of technology and communication that has self-organized during the event to provide coherent, relevant information outside the traditional information providers during a disaster. The spontaneous response to disaster was particularly acute after Hurricane Katrina in the US, coupled with Internet and mobile applications outside the traditional structure of information dissemination and emergency management [33]. Additionally, these events reinforce emergency management as a community activity that is local and linked to national level priorities [45].

The combination of internet technologies, the open source movement, the rise of citizen journalism (or blogging), and mobile technologies create a network of various participatory activities. Disasters have provided a unique trigger that have consolidated technological advances in concert with democratizing influences operating outside the traditional brokers of information and aid. A further development of the ODRC is the use of GIS to provide rapid information about locations that are experiencing the disaster.

## Internet GIS and Disasters

Understanding and managing disasters calls for specialized data, data networks, and information processing methods and technologies for GIS, disaster simulation, planning and risk assessment [35]. The information for and about a disaster are not necessarily readily available ahead of time due to highly dynamic and uncertain conditions [29]. However, during and post disaster reveals high levels of access to, pooling and sharing of digital resources, skills, and capabilities through the creation of novel and innovative socio-technological networks ([20, 29, 34].

There is a rich literature that addresses the use of GIS and disaster management. This research includes an overview of GIS and public safety [1], GIScience and applications for emergency response particularly after the September 11 World Trade Center attack [8], the development of disaster recovery networks [30] and local governmental responses to disaster using GIS [15]. The integration of the Internet with GIS applications has been applied to such areas as 3-D real time emergency response [27], serving maps on the internet for emergency escape routes [16], and mobile GIS and digital video for urban disaster management [32]. This literature focuses on emergency responders, practitioner communities, and governmental agencies. The role of people - activists, local communities, technologically savvy users, and citizen journalists, using and collecting geospatial data and developing GIS applications has been largely overlooked.

The intersection of formal and informal social networks creates the ODRC that is increasingly using Internet GIS as another form of communication about the disaster. Internet GIS develops due to the capacity for creative innovation, flexibility and interactive exchange in the spontaneous socio-technological networks that develop, access to open source code, and availability of digital data. The integration of mapping, global positioning systems, satellite imagery, and interactive geographic information systems provide important opportunities for developing and sharing information and techniques. In some instances, special licensing arrangements, “technological gift giving” result in innovative sharing and development opportunities during disasters [31]. Other innovative developments take best advantage of online resources. Mashups, the mixing of hybrid Web applications from multiple sources but appearing seamless to end users, combine satellite imagery with maps and geospatial data providing local information using the open Application Programming Interfaces (APIs), for example, of Google Map and Google Earth [31].

Development of internet GIS exemplifies its multi and interdisciplinary nature, integrating computer science, communications, geography, emergency planning, and disaster management. Internet GIS for disasters is composed of the following characteristics:

- Networks:
  New, informal, and often unofficial socio-technological networks form – the ODRC. This socio-technological network represents the recursive connectivity between people and technology, between centralized groups and individual users, and between private and public sectors [26]. These networks combine social groups through technological capabilities linking groups of informal Internet bloggers and networks of private companies, governmental agencies, and NGOs. Additionally, ad hoc technical networks have developed that allow for the intersection between wireless and wired infrastructures, mobile technology, and early re-establishment of Internet and computing facilities after a disaster [3].
- Data:
  GIS analysis converts raw data (eg., satellite image) into usable and relevant information. Specific, accurate, and targeted information on local conditions have the potential to be provided to workers and victims in a timely fashion. Satellite imagery is made available and processed via licensure arrangements and networks to provide information. Information may include evacuation routes, shelters, or locations of evacuees. Satellite imagery can depict the geographic extent of the damage and extent of damages done to the built environment, depending on availability, cloud cover, and resolution coupled with GIS overlays of roads, buildings, and infrastructure.
- Delivery:
  Data development of products is accomplished via innovative licensing structures with software vendors and data providers as well as GIS users. Downloadable data is available from data providers and widely distributed to aid workers, emergency responders and decision makers. During emergencies, software vendors have worked closely with emergency responders to develop necessary data for the emergency event. Due to the hierarchical response of disaster response and the traditional channels for information use and dissemination, not all of this information is necessarily made available to the general public. The ODRC has created mashups, webpages, wikis and blogs to link people to information sources and provide information in the form of maps when traditional avenues have failed [7, 31].
- Product:
  Numerous products have been developed from Internet GIS: multifunctional databases, open source GIS applications, specific software applications, services running on the Internet, information, and maps. Integrated and relational databases are the backbone of Internet GIS. Internet GIS for disasters provides technology-based enhancements to further develop multifunctional databases [32]. The development of multifunctional databases allow for a variety of applications such as revenue collection, natural resource protection, land-use planning, and infrastructure planning as well as disaster planning [32]. Open source code is critical to the ODRC for the development of online GIS products for disasters because the user to can change, use improve, and redistribute the software. In times of emergency and disaster, this ability is particularly important for innovative solutions and applications. Open source software can be free, and can be easily customized to meet a variety of end-user requirements. Additionally, the Open Geospatial Consortium, made up of companies, government agencies and universities, support interoperability across platforms, software, and data types to enhance functionality. Different products have been produced by both formal and informal groups with different audiences in mind. Visual outputs in the form of maps provide shelter locations and emergency evacuation routes at the local scale for disaster victims. Satellite images of coastlines before and after the tsunami and Hurricane Katrina provided important regional and temporal perspectives for scientists studying the physical nature of the disasters.
- Interactivity:
  Interactive capabilities allow for querying of and adding to databases. Some webpages have created interactive capabilities, such as the ability to download or add data. Exactly how Internet GIS is being used as an interactive medium is critical to understanding future use for disaster management [36].
- Connectivity:
  Embedded in this discussion is the assumption that people have access to the Internet. Connectivity is a crucial aspect of Internet GIS, specifically and of Internet access generally [28]. Other types of connectivity are also important: the extent of support for sharing, exchange and access of data, information and products as well as creating greater connectivity and interaction between the various tiers of the ODRC.

Coupling Internet GIS with community activism has resulted in the ODRC. This community reveals some of the inherent problems with cyber-disaster management, but also serves to create new opportunities for collaboration and information exchange. Collaboration and coordination between governmental agencies, humanitarian organizations, and private companies remain problematic due to conflicting missions, data security issues, and inadequate funding of emergency response technologies [33]. Understanding the capabilities of the ODRC offers an important opportunity for integration of local perspectives and skills to augment agencies stretched to capacity by multiple disasters and inadequate funding.

## Global and Regional ODRCs

The first tier of the ODRC is made up largely of global and regional consortiums that represent the formal networks addressing disaster education and awareness and training for humanitarian aid workers, local authorities, field personnel, and project managers. Most of these sites provide access to technical advice about disaster response, training opportunities for GIS disaster applications, direct access to satellite imagery and technical help in processing digital data, access to reports, and links to other information portals. There consortiums provide services for the development of GIS and remotely sensed products with regard to specific disaster events, how to collect and process data at the local. These products are made available at their websites for viewing and downloading final products. Generally, these websites do not provide interactive querying capability, although the Tsunami Gallery sponsored by Space Imaging (and which is linked from several global sites) allows one to zoom in on an image for a closer view. Many provide their latest satellite images and maps of the disaster event on line.

Some examples include, UNOSAT (www.unosat.org) provides the international community with “updated and accurate geographic information and to universalize access to satellite imagery [49]. Relief Web (www.reliefweb.int) provides information (documents and maps) on humanitarian emergencies and disasters and is designed specifically to assist the international humanitarian community in effective delivery of emergency assistance. The Global Disaster Information Network is a voluntary, independent, self-sustaining, non-profit association and seeks to provide “the right information, in the right format, to the right people in time to make the right decisions” (www.gdin.org). It is a consortium of industry, government, education and NGOs that develop unique information sharing procedures that augment the existing system and develop new disaster information management methodologies.

Regional efforts include training and information dissemination as well as early warning projects. Regional efforts can use the Internet but often in very different ways. The Asian Disaster Reduction Center (ADRC) (http://www.adrc.or.jp/) builds upon a formal network between government, private interests and industry to develop a state-of-the-art Internet GIS. The RANET project (www.ranetproject.net) uses Internet technology to disseminate early warning information, satellite imagery, weather, and climate data to rural areas. The RANET project seeks to share information, but builds on the existing capacity of the communities it serves where an Internet GIS may not be the most appropriate technology due to factors such as bandwidth, literacy levels, and data availability. The application of appropriate or best-fit technologies is a critical aspect of GIS and Internet applications for disaster management in developing countries taking best advantage of existing socio-technological networks.

This first tier of the ODRC provides several important services: their websites act as a resource base for humanitarian organizations in providing clearinghouses for information, reports, data, resources, and training opportunities. They provide information and maps on ongoing disasters both to the general public and NGOs using GIS data, satellite imagery, and information collected from the on-sight first responders. Training specifically provide tools and techniques that link local observations with satellite imagery and GIS data layers to develop disaster responses to create the basis for “people as sensors” approach, individuals on the ground who respond to the disaster to create important maps and GIS analysis. Most of these sites are hyper-linked to each other, creating a global network of information and mechanisms to provide the latest information on an on-going disaster. These sites archive this information creating an historical record of global disaster events. These products are often posted during and after disasters using the Internet technologies for data dissemination.

## People as Sensors: Cyber-community-based Approaches to Participation and Information Dissemination

The ODRC routinely form in cyberspace following disasters. One area where members of the cyber-community share a common interest is in getting money and aid to disaster victims in a secure and efficient manner [23]. Both the hurricane and the tsunami brought together a combination of forces. First, the public was able to use the Internet and the online news media as resources to learn the latest news about the disasters, including detailed personal accounts. Second, the online news media featured prominent links to Web sites for charities that were equipped to deliver money and aid quickly to the stricken areas. Third, the public felt confident enough in the security of Web sites to donate online using their credit cards. And fourth, the public remains connected to the events through continuing coverage allowing people to track recovery and reconstruction efforts. These forces combined to become a powerful motivator of individuals to donate to disaster relief, help explain the tremendous growth in online giving and enhanced the need for community-based perspectives. Community perspectives are of particular importance in targeting aid to meet needs. After the Indian Ocean tsunami and Hurricane Katrina, people were inundated with information and media coverage. Donations and aid reached extraordinary levels resulting in inappropriate donations of clothing and food and the need to track government pledges of support [14].

A further outcome of the Indian Ocean tsunami and Hurricane Katrina is the recognition of the “first responders of the wired world” [7]. These first responders are a crucial part of the ODRC and contribute through innovative uses of existing technology: blogs, message boards and web portals. Taking advantage of the combination of the Internet's technological capabilities, one of the quickest sources of news about both the tsunami and hurricane came from blogs, a form of personal diary in cyberspace [25]. These blogs posted first-hand accounts, personal observations, photos, and videos in order to share their experience and information (www.punditguy.com). Message boards, such as NOLA.com (http://www.nola.com/forums/searching/index.ssf), were critical in providing information about shelter locations, family tracing, and missing persons. Wiki software was used as an organizational tool to create web portals (http://katrinahelp.info/wiki/index.php) to web pages such as those identifying immediate shelter needs (ShelterFinder) and family tracing (PeopleFinder). PeopleFinder was a volunteer effort to create a consolidated database of missing people built outside the traditional, centralized institutions (i.e., FEMA, Red Cross) [7]. Adopting the open source prerogative of sharing software code, bloggers created a data standard: People Finder Information Format (PFIF), has been utilized in other disaster events. This output exemplifies what Geilhufe has termed “social source” – the coupling of nonprofit technology with the open source movement explicitly for social missions [13].

These activities operate at the local level and are in immediate response to the disaster event. Socio-technological networks can be informal and unofficial, with the development of numerous web logs, web portals, and message boards. The acceptance and expansion of on-line giving strengthens the cyber-community, creating a vested interest in the disaster event, linking people from disparate geographies [24]. These resources attempt to distribute appropriate, accurate information in a timely fashion and in some instances, in real time. Spatial data and information is implicit at this level rather than explicit as in Internet GIS applications. Spatial information is largely identified in questionnaires to identify where people were last seen, address information, or shelter locations.

## Opportunities for Development of ODRC: The Third Tier

Internet GIS for disasters or digital disaster systems are rapidly evolving in the face of ongoing, back to back, disasters. An important aspect of these systems is the recognition of the importance of satellite imagery and the critical need for access, processing, interpretation, and use of these images for disaster response and management. The products of GIS for disaster can be found on several sites, many of which are developed by government agencies and industry partnerships. What is more promising for the ODRC are such applications that allow for interactive use of satellite imagery with local information and knowledge.

- Google Earth and Google Map:
  Google Earth and Google Map provide the most promising arena for Internet GIS by the ODRC. Google Earth (http://earth.google.com/index.html) provides access to on line satellite imagery. The program must be downloaded to view the imagery – but most imagery is available in Google Map. This imagery has been used to develop products of damage assessment for Hurricane Katrina and was used to share tsunami images.
  Google Map (http://maps.google.com/maps/ms?f=q&hl=en&geocode=&time=&date=&ttype=&ie=UTF8&om=1&msa=0&msid=114250687465160386813.00043d08ac31fe3357571&ll=32.990236,-116.732483&spn=1.105782,1.757813&z=9&mid=1193110631) has been used to develop maps of shelter locations and fire updates during the 2007 wildfires in California. Maps can be overlaid with satellite images of the same location.

A further development in response to the multiple disasters has been the formation of volunteer organizations that provide hands-on expertise to develop location-specific, GIS applications. These organizations form in cyberspace to solicit assistance in times of need development and implementation of socio-technological networks for disaster response:

- GISCorps: http://www.giscorps.org/
  Operating under the auspices of the Urban and Regional Information Systems Association (URISA), GISCorps coordinates short term, volunteer GIS services to underprivileged communities worldwide. They provide GIS services that include: needs assessment and strategic planning, technical workshops, database modeling, disaster management, and remote sensing processing and interpretation. GIS allows relief agency staff to obtain critical information about how humanitarian support efforts are progressing to ensure appropriate response agencies are acting in a coordinated and efficient manner. Once in the field, the coordination can continue as new data can be added and disseminated via wireless applications and Internet/Intranet connectivity.
- Mercy Corps: http://www.mercycorps.org/
  Mercy Corps has created the Geospatial Relief & Development Team. A volunteer base of more than 50 GIS and remote sensing professionals in the Pacific Northwest has mobilized to apply geospatial technologies to expedite the flow of aid and accelerate recovery. They seek to establish a Non Governmental Organization (NGO) geospatial coordination team to reduce redundant efforts in emergency mapping, increase efficiency, detect change, transfer knowledge and provide a geospatial data repository for all NGOs in collaboration with the United Nations Geospatial Initiative throughout all phases of recovery.
- MapAction (http://www.mapaction.org/)
  Based in the UK, MapAction is an NGO dedicated to providing time-sensitive information during a disaster. They integrate geospatial technologies (GIS, GPS, and RS) to create maps developed by a cadre of volunteers. In addition, they provide training programs in developing countries.

A further component of Internet GIS for disasters is the rise of citizen GIS, participatory GIS, and civic web mapping. Existing systems managed by local and federal agencies were not strong enough to handle disasters of the magnitude of the twin hurricane events. As local and federal agencies struggled to respond to Hurricanes Katrina and Rita in 2005, two software engineers created Scipionus.com – a Google map of affected areas with site-specific comments provided by numerous individuals. Online grassroots disaster response demonstrated the power and speed in which people could disseminate information. Creating mash-ups by seamlessly combining data from other sources with Google maps do not necessarily require geospatial skills and GIS expertise.

- Hurricane Information Maps: www.scipionus.com
  Using Google's free API (application programming interface), this site presents information using Google maps with other data creating a “mash up” or a mash up of programs [9]. These maps were designed for people affected by Hurricanes Katrina or Rita who were trying to find information about the status of specific locations affected by the storm and its aftermath. This site explicitly asks for updated information and provides instructions for how to add new locations and their attribute information.

The combination of mapping tools, geospatial data, and web applications provide an avenue for empowerment for people to self-organize in response to disaster. While emergencies vary widely in scale, severity, and duration, they are inherently local. Oftentimes, information that is needed from a GIS for immediate emergency response are inherently simple – not requiring complex analytical procedures but reliable and adequate data. Individuals with local roots (one of the Scipionus creators was from New Orleans) while in a remote location (Austin, Texas) could devise an application that addressed the disaster [39].

## Problems and Caveats

Disasters often reveal the divides in society. Hurricanes Katrina and Rita revealed the racial, class, and technological divides in Louisiana, Mississippi, and Alabama. While satellite images of the Louisiana coast were being posted hours after the storms hit, people were still waiting for rescue days later. The disaster digital divide is evident worldwide. Accessibility to a reliable computer network and internet connections is dependent upon an overall, well-developed infrastructure. Knowledge of data availability and access to copyrighted and classified data is another issue evident in developing countries as well as developed countries. After September 11, 2001, access to infrastructure data across the US was curtailed. Most developing countries are technologically vulnerable and have problems with technical security. They often lag behind in technical expertise, computer literacy, and often, basic literacy.

Developing countries have complex political, social and economic environments where Internet GIS will be difficult to facilitate. For example, the Kashmir earthquake (2005) revealed the limitations in terms of access to sites and timely response that are evident when disaster strikes in remote locations far from infrastructure. Basic GIS data layers are not available and processed satellite images revealed little in the way of damage assessments. The ubiquity and power of information and communication technologies that many take for granted are not evidenced by their actual performance in the face of catastrophe [44].

The ability to zoom in and zoom out of maps and images masks the role of scale in examining disasters through digital databases. Such databases are only as good as the data. The availability and accessibility of the right data for the specific application have continued to plague the development of GIS databases. In large unprecedented events, the maps and information that are needed for doing the groundwork at the local scale immediately after the event are often not available [31]. The availability of such data is location-specific, dependent upon the country, state, county, province, or village. Even if such data were available, it often does not provide enough information about the social landscape and alternative representations of the disaster which must come from field-level data [17].

Internet GIS coupled with satellite imagery allow for the remote construction and representation of a disaster. This can be used to both empower the grassroots as in the case of Scipinious.com or can be used to narrow the focus of disaster recovery. Most digital views of the disaster focus on the location of immediate impact using the best available data. This best available data increasingly includes satellite imagery captured at spatial scales that do not reveal the entire disaster story. GIS and remote sensing are high technology solutions that can filter out local equity and social welfare issues [17]. Additionally, disasters often include relocation and movement of large numbers of people to another area, the establishment of refugee camps, and the infusion of different groups of people into new areas that may not have the necessary infrastructure to support them. Such receiving areas are seldom included in geospatial analysis of disaster recovery. This was not necessarily the case in Hurricane Katrina where relocation of victims became part of the larger national disaster and emergency management fiasco [51].

Regardless of where one is in the world, the flow of information has increased in complexity [12, 38]. Even remote locations have multiple means of gathering information from mobile technologies, such as cell phones and text messaging. The Internet has numerous sites that exist for disaster information. There are multiple sites that post information from multiple sources. Many sites provide information that are redundant and outdated. With individuals posting grassroots information as well as NGOs and governmental agencies, how will one know where to get the best information? How will the humanitarian community, GIS users, the public choose between the different outputs and technologies?

An important caveat is that use of the Internet is predicated upon access. Access implies appropriate infrastructure [28]. While access to the Internet is increasing exponentially, there is an uneven distribution of network connections and access points. Additionally, the current record with regard to Internet GIS for disaster management is largely fragmented, uncoordinated, and reactive. However, the web-based products create expectations that these results will make a difference for the next disaster.

Information that is necessary to respond effectively to a disaster is not easily identified ahead of time, nor is it ever complete. However, conclusions need to be drawn and decisions made with regard to action and mitigation. Information assessment for disaster management need to be closely examined to determine if such databases and GIS products are really meeting the needs of the people. The ODRC demonstrates the need for local, technological responses that are coupled with traditional mechanisms of emergency response from government and aid agencies. Clearly, information is available, however, are adequate policies and procedures in place for information management for disasters? Do we have the necessary multifunctional databases to analyze and assess disaster? What data is not included? What data is not in digital format that is important for disaster management? In developing such products, in what ways are funding and support diverted from other disaster management activities?

## Future research

Disaster response includes the traditional government and NGO responders and due to technological breakthroughs, a wide range of participants from the public arena. These publics coupled with traditional responders create new, unique, and informal networks to contend with disaster in the digital environment. Some progress has been made in getting information where it is needed in a timely fashion. This has occurred due to cooperative efforts to share information, expertise, and resources. However, impediments due to the digital divide, access to technology, training and education as well as appropriate infrastructure inhibit Internet GIS development.

Several actions need to take place at both the formal and informal level. At the formal level or first tier of the ODRC, meaning government and aid organizations, the following activities need to occur:

- Establishing standards, protocols for practice and for data collection for disaster management. The Electronic Records Management E-Gov Initiative, the Federal Geographic Data Committee, and International Organization for Standards have contributed much to further seamless digital exchange and compatability. Such efforts need to be extended to protocols for disaster management.
- Building multifunctional databases. Multifunctional databases require bridging information gaps. For example, cadastral databases are designed for land registration and taxation purposes that are not easily adapted to natural hazard loss estimations [32].
- Building and maintaining early warning systems while ensuring the necessary institutional support and recognizing that disaster warning is a core business of government. The 2004 tsunami revealed the need to develop not only the use of information communication technologies, but also to ensure the development of institutions that can support them [44].

At the informal level, the ODRC that includes first responders, citizen groups, activists, and the local community, the following actions need to occur:

- Understanding and identifying the role of emergency management agencies and humanitarian aid workers with regard to geospatial technologies and disaster and information dissemination for local communties. Increasing skills in information systems for emergency managers and humanitarian aid workers to better understand the role of data collection and information for emergency management [6].
- Identifying the role of and relationship with local, community-based organizations and efforts to integrate community-based solutions for disaster management. Emergency management is a community activity; most first responders are local. Developing training for community-based emergency data collection would have immediate benefits during a disaster for localities [21, 45, 46]. Developing drills for emergency response that include GIS applications and rapid response assessments and analysis.
- Recognizing the role of innovative and serendipitous activity to fill gaps that government agencies can not, do not, or will not. Several grassroots, open source development projects resulted from Hurricane Katrina: damage assessments reporting capability, missing persons databases, and sharing of reporting on calls for help. The formation of GIS Corps is the result of understanding the need for technical expertise in disaster areas. Taking best advantage of open source solutions for disaster management technology and technological gift-giving is an emerging area for disaster management [31].
- Further development and resources for Mobile GIS activity. Integration of GIS and GPS that will further the establishment of real time data collection and understanding the challenges it presents for first responders as well as the ODRC [5].

Developing partnerships between the formal and informal sectors to develop better digital disaster systems:

- Identifying and understanding the role of “unmapped” places for disaster management. Building on projects such as the UN Living with Risk [50] and the World Bank's Disaster Hot Spots [11], urban areas in developing countries have been identified as particularly prone to disasters due to land use practices and higher densities of population. Periurban areas surrounding Mexico City, Sao Paulo, Rio de Janeiro, Kuala Lumpar, Manila are often unmapped with little or no information making disaster management particularly difficult [32].
- Assessing the viability of Internet GIS for disaster to provide help to those who need help. Remote construction of a disaster via GIS and remote sensing focuses on the disaster itself outside the context of the socio-political environment. Ensuring adequate field-based data and community-based information is crucial to understanding the disaster, the victims, and its aftermath [17].

## Conclusion

Braudel wrote, “Technology is only an instrument and humans do not always know how to use it” [4]. Technological advances often outpace human adaptative responses and social structures [12]. While advances have been made in using GIS for disaster management, we are still learning how to best use the technology for application and analysis. Hence, each disaster becomes a teaching moment to better understand how to use Internet GIS for disasters. Each disaster also reveals responses that reproduce power relations [19].

Important lessons learned from recent disasters include the development of new networks for data exchange, innovative licensing arrangements to facilitate use of software and data, and development of novel applications of open source code. Technological integration has led to the emergence of mobile GIS building and enhancing existing models for developing data capture of real time data using GPS units. The role of local communities and the ODRC has also been identified as key to developing disaster management strategies during pre and post activities. The importance of recognizing local solutions based on best-fit technologies and options for self-mobilization is crucial in many developing countries. Further, the spontaneous networks that developed after major disasters were in part influenced by the Internet that provided the environment for an online disaster response community [24].

There have been major changes in the nature and scope of crises, emergencies, and in the types of threats to the public that include terrorism, war, and human made disasters as well as natural disasters. Effective communication, such as effective GIS activities, must begin long before the event erupts and continue long after the immediate threat has subsided. Internet GIS for disaster management reveals the power of intersecting networks to create new organizational and networking arrangements for addressing disaster and valuing the role of “people as sensors” – to collect information about their locality and disseminate to those in need. It reveals the relationship between geography, communication, and technology. However disaster management that includes Internet GIS must include an evaluation that demonstrates its utility and success. The grim reminder of helpless victims stranded on roof tops after Hurricane Katrina is juxtaposed with the satellite images of the Louisiana coastline. The critical requirement is to provide help those who need help and to discern the role of Internet GIS in this capacity.
